# Safety of Antithyroid Drugs in Avoiding Hyperglycemia or Hypoglycemia in Patients With Graves’ Disease and Type 2 Diabetes Mellitus: A Literature Review

**DOI:** 10.7759/cureus.41017

**Published:** 2023-06-27

**Authors:** Yu-Shan Hsieh

**Affiliations:** 1 School of Nursing, National Taipei University of Nursing and Health Sciences, Taipei City, TWN; 2 Department of Research, Taipei Medical University Hospital, Taipei City, TWN

**Keywords:** hyperthyroidism, thyroid disease, blood sugar control, glucose stability, carbimazole, methimazole, propylthiouracil, antithyroid drugs, type 2 diabetes mellitus, graves' disease

## Abstract

Graves’ disease (GD) may increase the difficulty of glucose control in patients with type 2 diabetes mellitus (T2DM). Therefore, selecting a drug with limited blood glucose side effects is an important issue in patients with T2DM and GD. Antithyroid drugs (ATDs) including propylthiouracil (PTU), methimazole, and carbimazole are commonly prescribed for the treatment of GD. Here, we review and summarize the literature from the last 10 years and discuss the effects of current ATDs used for GD for blood glucose control in patients with T2DM. A search of the literature published between January 1, 2012 and December 1, 2022 was conducted using three major medical databases: Google Scholar, Ovid Medline, and Scopus. An initial search was conducted on PubMed using the MeSH terms “propylthiouracil,” “methimazole,” “carbimazole,” and “hyperglycemia” or “hypoglycemia” in academic databases. All articles included “Graves’ disease” and “type 2 diabetes mellitus” in the title.

Based on the results of previous studies, the hyperglycemic and hypoglycemic side effects of ATDs can be explained by several possible mechanisms. The most widely accepted hypothesis is that sulfhydryl group drugs (e.g., methimazole and carbimazole) cleave the disulfide bond of insulin and enhance its immunogenicity, resulting in hypoglycemia. Moreover, some reports have indicated that methimazole is associated with hypoglycemia; therefore, if the patient has a history of autoimmune diseases, it is necessary to consider whether to change drugs or actively track the production of autoimmune antibodies. In non-diabetic and diabetic patients with GD, the hyperglycemic and hypoglycemic side effects of PTU (on glycemic variation) were less than that of thiamazole. However, as relatively few reports have investigated the side effects of blood sugar changes, further research is necessary to confirm these effects. In addition to autoimmune diseases, drug side effects may need to be considered. These findings provide considerations for clinicians to select more appropriate ATDs for patients with GD and T2DM, and implement improved care guidelines.

## Introduction and background

In clinical practice, patients with multiple endocrine diseases who take multiple medications commonly experience many side effects. Antithyroid drugs (ATDs) can cause unstable blood sugar in patients with thyroid disease. Graves’ disease (GD) is an immune disorder that results in hyperthyroidism [1]. GD is the most common form of hyperthyroidism, accounting for approximately 60%-80% of cases of thyrotoxicosis [2,3]. Factors known to be associated with abnormal glucose tolerance include an abnormal metabolic rate, glucose absorption, and endogenous gluconeogenesis, all of which can be affected by thyroid hormone levels [4]. Additionally, GD may increase the difficulty of glucose control and is associated with complications such as diabetic ketoacidosis in patients with concurrent type 2 diabetes mellitus (T2DM) [5].

GD is more common in patients with T2DM than in the general population and can influence blood glucose control. Goiter has also been recognized as a risk factor for thyroid disease in patients with diabetes mellitus (DM) [6]. Moreover, free thyroxin (FT4) level in patients with uncontrolled GD is a key contributor to their increased glycemic variation [7]. Among the types of DM, T2DM accounts for approximately 90% of all cases. Both hyperglycemia and hypoglycemia are important complications of T2DM, especially hypoglycemia. A previous study showed that the incidence of severe hypoglycemia ranged from 0.7 to 12 episodes per 100 person-years [8]. Hypoglycemia is one of the most common events requiring emergency department care or hospitalization. The Centers for Disease Control and Prevention (CDC) in the USA reported that there were 235,000 emergency department visits for hypoglycemia in 2016 (10.2 per 1,000 adults with diabetes) and 224,000 for hyperglycemic crises (9.7 per 1,000 adults with diabetes) [9].

In patients with T2DM, stabilization of glucose variability is an important issue in the drug treatment of GD. Severe hypoglycemia and hyperglycemia can lead to loss of consciousness and may become life-threatening. Both iatrogenic hypoglycemia and hyperglycemia are known causes of complications, requiring emergency care or hospitalization in patients with T2DM [10]. Increased fasting and postprandial blood glucose levels could improve with the normalization of thyroid function [11]. However, previous studies have reported the occurrence of hypoglycemia in patients with GD who were treated with methimazole [12,13], carbazole [14], and propylthiouracil (PTU) [11].

Selecting an optimal strategy to treat GD is an important issue in patients with GD and T2DM. The most commonly prescribed ATDs for the treatment of GD include methimazole, carbimazole, and PTU. Herein, we review and summarize the literature from the last 10 years and discuss reports of the effects of ATDs currently prescribed for GD on blood glucose control in patients with T2DM.

## Review

Survey methodology

A literature search was conducted using three major medical databases, including Google Scholar, Ovid Medline, and Scopus, including articles published between January 1, 2012 and December 1, 2022. An initial search was conducted on PubMed using the MeSH terms “propylthiouracil” “methimazole,” “carbimazole,” and “hyperglycemia” or “hypoglycemia” in academic databases. All articles had to include “Graves’ disease” and “type 2 diabetes Mellitus” in the title. The references were checked, and the titles and abstracts of the references were searched to identify studies that met the following inclusion criteria: (1) participants who had GD with T2DM; (2) quantitative or qualitative measure of the glycemic variety; and (3) reported clinical research (case report, case series, observative study, or clinical trial). After locating the literature that met these criteria, additional material was identified and examined, before being rechecked by the author and endocrinologists involved in this study. The International Prospective Register of Systematic Reviews (PROSPERO) registration number is CRD42022366413 (October 2022).

Shared mechanism of GD and T2DM in glucose variability

**Figure 1:**
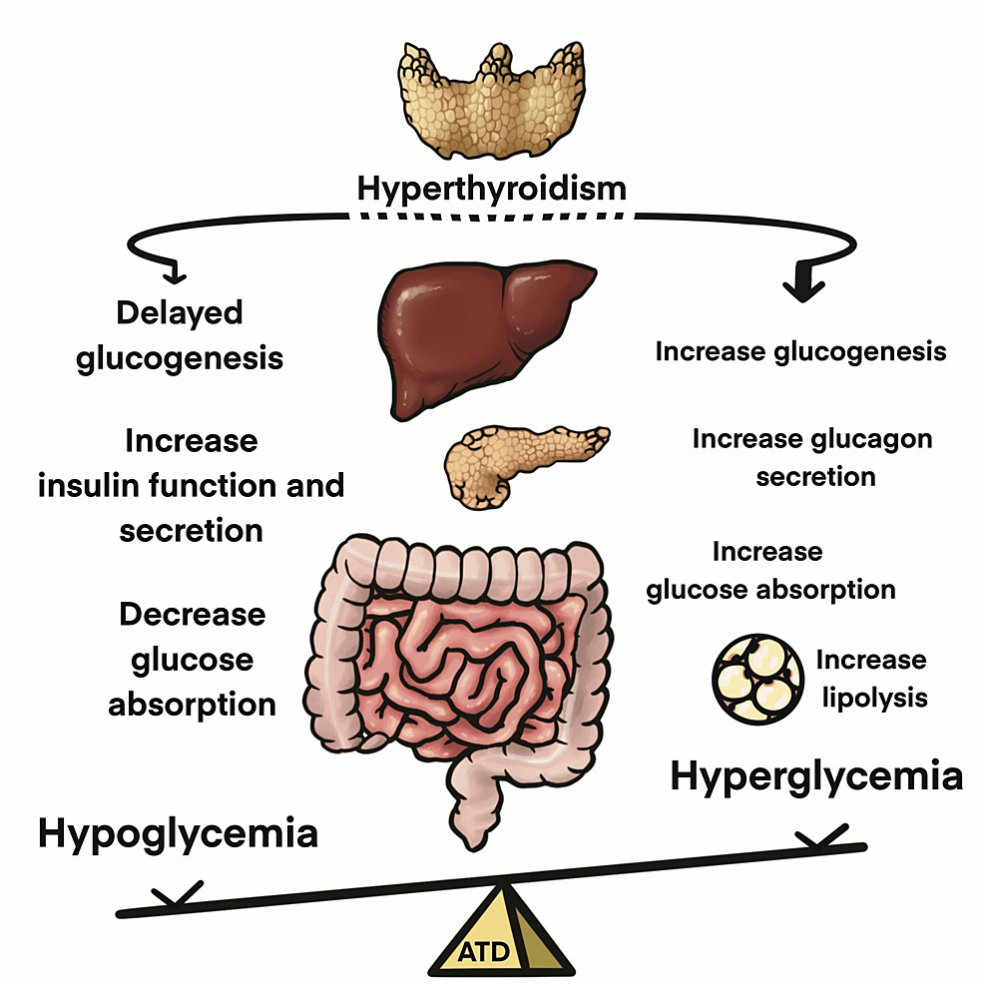
Both hyperglycemia and hypoglycemia may occur in patients with T2DM with hyperthyroidism, including those with GD. Hyperglycemia may be more commonly reported due to hyperthyroidism; however, the side effects of ATDs may aggravate the incidence of hypoglycemia ATD: Antithyroid drug, GD: Graves’ disease Image created by Yu-Shan Hsieh and Hsiao-Han Chang.

As per previous studies, elevated thyroid hormone levels affect glucose metabolism by modulating gluconeogenesis, glucagon secretion, glucose absorption, and lipolysis (Figure 1).

Modulation of Glucogenesis

Thyroid hormones can stimulate insulin-dependent glucose uptake, gluconeogenesis, and glycogenolysis in thyrotoxicosis [4]. Severe hyperthyroidism may influence the secretion of insulin [15], increase gluconeogenesis [16], and affect carbohydrate metabolism, leading to hyperglycemia [17]. However, severe hyperthyroidism has also been reported to induce hypoglycemia due to depleted glycogen stores [18,19].

Modulation of Glucagon Secretion

Impaired glucose tolerance, insulin resistance, and insulin secretion have been reported in patients with hyperthyroidism [17]. Both Hu et al. [20] and Yoshikawa et al. [21] reported an increased fasting insulin level, suppressed blood glucagon levels, and high insulin resistance in the GD population.

Modulation of Glucose Absorption

Enhanced glucose absorption [22] has also been found in patients with hyperthyroidism. Previous studies have reported that elevated thyroid hormone levels not only abnormally enhance glucose absorption by increasing gastrointestinal motility [23], but also increase the hepatic expression of glucose transporter 2 (GLUT2), which stimulates the endogenous production of glucose [24].

Modulation of Lipolysis

Han et al. [25] reported that angiopoietin-like protein 8 (ANGPTL8) was increased in patients with overt hypothyroidism. Abnormally elevated levels of thyroid hormone have also been reported to be associated with increased clearance of lipolysis and cholesterol [26].

ATD-induced hypoglycemia

Insulin autoimmune syndrome (IAS), also known as Hirata’s disease, is a rare disease of immune-mediated hypoglycemia, which is characterized by hypoglycemia due to the presence of high levels of insulin autoantibodies (IAA). Previous studies have shown that ATDs can induce IAS, resulting in hypoglycemia. Approximately 50% of patients with IAS have some specific medication history, and more than 90% of the agents are sulfhydryl compounds such as methimazole, mercaptopropionic glycine, or glutathione [27]. Thus, we review and summarize the literature from the last 10 years (2012-2022) and discuss the effects of the ATDs currently used for GD (e.g., PTU, Carbimazole, and Methimazole) on blood glucose control in patients with T2DM.

PTU

In previous studies, hyperglycemia [11] and GD-induced IAS [28] were reported could be improved after changing the ATDs from methimazole to PTU. However, the improvement in hypoglycemia was not reported. However, in a case study of pyoderma gangrenosum with T2DM [29], PTU was reported to induce anti-neutrophil cytoplasmic antibodies and IgG antibodies, which could change the activity of neutrophils to mediate disease [30]. Based on the difference in chemical structure between PTU and methimazole, the reported side effects of PTU on glycemic variation were less than those of thiamazole in both nondiabetic and diabetic patients with GD.

Carbimazole

In 1982, an early study reported the occurrence of hypoglycemia as a side effect of carbimazole in patients with T2DM with hyperthyroidism [31]. However, in a case report of non-diabetic ketoacidosis due to hyperthyroidism, carbimazole was found to effectively improve the disease, without affecting blood glucose levels [32]. Another case study reported severe hypoglycemia in a patient with hyperthyroidism [33].

Methimazole

IAS has been reported to be induced by methimazole and carbimazole [34]. IAS is characterized by hyperinsulinemic hypoglycemia and elevated serum insulin autoantibody levels [35]. A previous study reported 60-69 years as being the peak onset of IAS [36]. However, in the last decade, IAS has also been reported in adolescents [37] and elderly individuals [12]. Although the cause of IAS remains poorly understood, the most widely accepted hypothesis is that sulfhydryl group drugs, such as methimazole and carbimazole, cleave the disulfide bond of insulin and enhance its immunogenicity in patients with T2DM and GD [38]. According to a previous study, the human insulin α-chain binds with high affinity to HLA-DRB1*0406. The SH group of methimazole could cleave the disulfide bond of insulin, leading the linear fragment on the α-chain exposed to the DRα-DRB1*0406 to bind it with high affinity; this interaction could increase the activation of insulin-specific T cells and the formation of IAA. The increasing IAA complexes result in elevated levels of circulating insulin, causing hypoglycemia [39]. IAS may occur secondary to GD, which is a common autoimmune disease in patients with T2DM. Autoimmune diseases, including IAS, may occur as a result of insulin treatment or GD, in which case, methimazole, and steroid treatment may improve hypoglycemia [12]. Thus, IAS may be associated with immunodeficiency or immune disorders in patients with T2DM [28].

**Figure 2:**
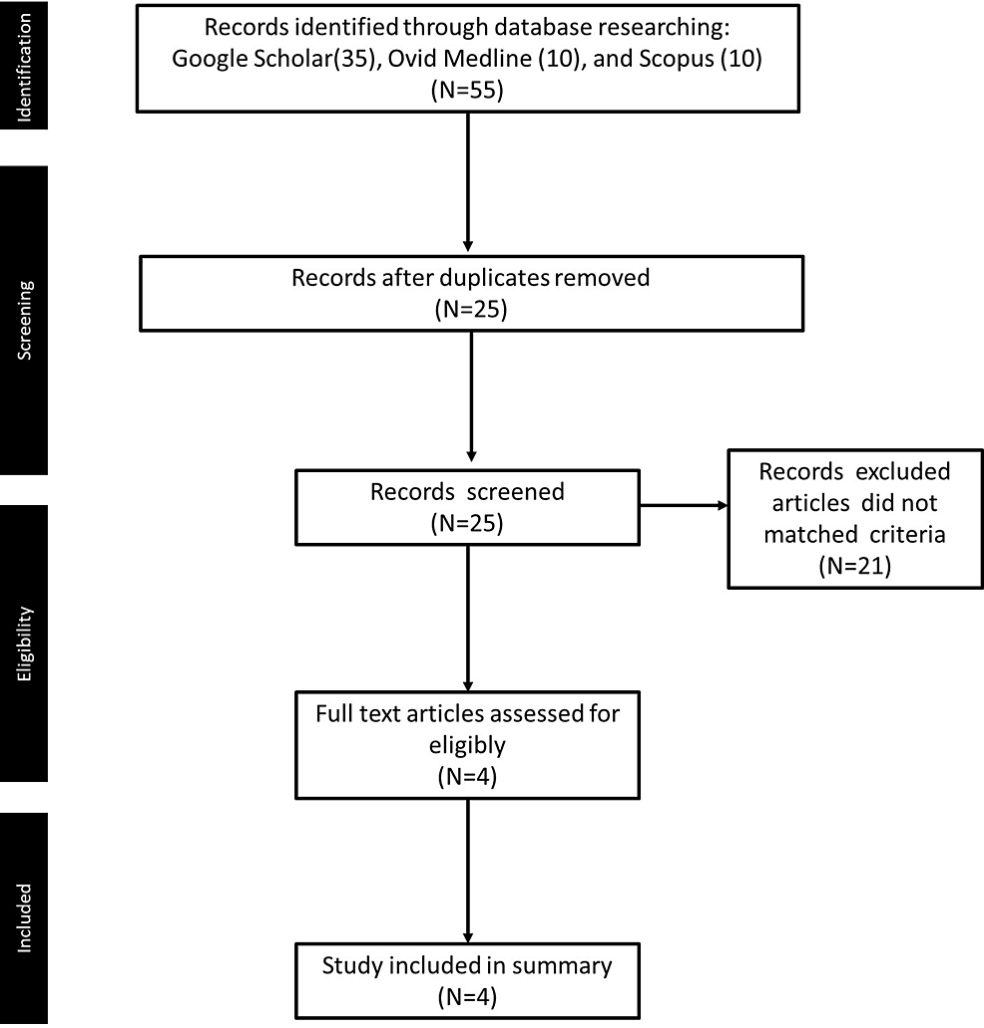
Preferred Reporting Items for Systematic Reviews and Meta-Analysis (PRISMA) flow diagram Created by searching the MeSH terms “methimazole,” “carbimazole,” and “hyperglycemia” or “hypoglycemia” in academic databases.

Although the cause of IAS is incompletely understood, previous research has indicated that patients with GD with gene Bw62/Cw4/DR4 carrying HLA-DRB1*0406 are at a higher risk of developing IAS from methimazole use [40]. Additionally, a previous study conducted in Korea found that non-diabetic patients possessed HLA-DRB1*0406 gene expression, which is strongly associated with methimazole-induced IAS, and may be associated with methimazole-induced hypoglycemia [41]. Furthermore, there have been reports of an increased incidence of hypoglycemia and IAS with methimazole treatment in patients with T2DM and GD [1,23,35]. Among the three commonly used ATDs, methimazole appears to have the highest risk of inducing hypoglycemic side effects in patients with hyperthyroidism. Although methimazole is a widely used ATD in the treatment of GD, its side effects, including IAS, should be considered as causes of hypoglycemia in patients with T2DM and GD. Therefore, we further investigated whether this phenomenon was also present in patients with T2DM and GD by searching the MeSH terms “methimazole,” “carbimazole,” and “hyperglycemia” or “hypoglycemia” in academic databases. The Preferred Reporting Items for Systematic Reviews and Meta-Analysis (PRISMA) flow diagram of the selection process is shown in Figure 2.

All articles had to include “Graves’ disease” and “type 2 diabetes mellitus” in the title. A summary of the clinical reports of methimazole-induced hypoglycemia in patients with or without T2DM in the last decade is presented in Table 1.

**Table 1:** Summary of reports of methimazole-induced hypoglycemia in patients with or without T2DM MTZ: Methimazole, PTU: Propylthiouracil

Reference	Patient characteristics	Treatment of GD	Treatment of T2DM	Therapeutic outcomes	
Gomez Cruz et al. [37]	16-year-old African male without T2DM	MTZ 20 mg TID	Not presented	PTU was substituted for MTZ	
Wang et al. [12]	85-year-old Chinese female with T2DM	Not presented	Oral antidiabetic drugs with insulin aspart 30R 12 U QD	Prednisone 20 mg and MTZ 10 mg QD	
Roh et al. [41]	53-year-old Korean female without T2DM	MTZ 30 mg QD	Not presented	MTZ was subsequently discontinued	
Roh et al. [41]	52-year-old Korean female without T2DM	MTZ 40 mg QD	Not presented	MTZ was subsequently discontinued	
Chen et al. [28]	76-year-old Chinese male with T2DM	Not presented	Insulin aspart 30R	Acarbose 50 mg TID was substituted for insulin	
				Prednisone acetate tablets 7.5 mg QD	
Chen et al. [28]	50-year-old Chinese male with T2DM	Not presented	Insulin aspart 30R 10 U QD	Acarbose 50 mg TID was substituted for insulin	
				Prednisone acetate tablets 5 mg BID	
Chen et al. [28]	40-year-old Chinese female with T2DM	MTZ	Not presented	PTU was substituted for MTZ	

ATD-induced hyperglycemia

In the past decade, some studies have reported that patients with GD may experience hyperglycemia with T2DM [1,23,35]. However, there is limited evidence of the direct association between ATDs and hyperglycemia.

Prevalence of hypoglycemia in patients with methimazole-induced IAS

Regulation of blood glucose may be complicated in patients with both T2DM and GD, and patients with T2DM may be at an increased risk of hypoglycemia due to differences in drugs. However, the presented studies highlight an increased risk in patients taking methimazole compared to other ATDs. Although many studies have reported that methimazole may induce IAS, IAS remains a rare cause of hypoglycemia. Takei et al. compared the prevalence of IAS in patients with GD without T2DM who were treated with methimazole or PTU as well as patients with untreated hyperthyroidism; as a result, they found that only 6.3% of cases had IAS among patients treated with methimazole [42], which is in agreement with the low incidence rate of IAS in Taiwan [43].

Based on previous studies, there are several possible mechanisms of hypoglycemia or hyperglycemia. Hypoglycemia can be attributed to either methimazole or carbimazole, which is converted to methimazole in patients with GD. Thiamazole-induced IAS was considered to be caused by an increase in α-lipoic acid (a reducing molecule) [44]. Another case study reported the occurrence of hypoglycemia and GD in a non-diabetic patient with the DRB1*0406 gene, in whom the total insulin normalized and no other episodes of hypoglycemia occurred following discontinuation of methimazole and prescription of PTU [45]. HLA genotypes are considered to have a strong association with IAS. Polyclonal insulin antibodies, which are observed in most cases of IAS, are strongly associated with HLA-DRB1*0406, DQB1*0302, and DQA1*0301 [46]. Among HLA genes, the HLA-DRB1 gene can bind to insulin-derived peptides and is cleaved by methimazole, which stimulates insulin-specific proliferation of T cells [47]. HLA-DRB1*08032 is another known risk factor for agranulocytosis induced not only by methimazole but also by PTU [48].

Methimazole is reported to be associated with hypoglycemia in patients with immune dysfunction [49]; therefore, if the patient has a history of autoimmune diseases, it is necessary to consider whether to change drugs or actively track the production of autoimmune antibodies. A lower therapeutic dose of glucocorticoids, including prednisone, is often reported [28,37,50]

## Conclusions

In conclusion, PTU's hyperglycemic or hypoglycemic side effects on glycemic variation were less than those of thiamazole in non-diabetic and diabetic patients with GD. Based on these results, PTU is considered to have less hyperglycemic or hypoglycemic side effects. However, there remain relatively few reports on side effects impacting blood sugar, and further research may be needed to confirm these effects. Moreover, if patients develop spontaneous hypoglycemia during the use of methimazole, the possibility of IAS should be considered and treatment with thiamazole should be avoided because of the potential for hypoglycemic side effects. If the patient has a history of autoimmune diseases, a change of drug or active tracking of the production of autoimmune antibodies is warranted. These findings provide key considerations for clinicians to select more appropriate ATDs for patients with GD and T2DM and implement more appropriate care guidelines.
